# Epidemiological characteristics of leprosy during the period 2005–2020: A retrospective study based on the Chinese surveillance system

**DOI:** 10.3389/fpubh.2022.991828

**Published:** 2023-01-11

**Authors:** Xiang Li, Guangjie Jin, Jing Yang, Yunhui Li, Pingmin Wei, Lianhua Zhang

**Affiliations:** ^1^Department of Epidemiology and Health Statistics, School of Public Health, Southeast University, Nanjing, China; ^2^Department of Chronic Infectious Disease Control and Prevention, Jiangsu Provincial Center for Disease Control and Prevention, Nanjing, China

**Keywords:** leprosy, Jiangsu, epidemiology, GIS, spatio-temporal analysis

## Abstract

**Background:**

Jiangsu Province is located in the Yangtze River Delta region, with a total area of 107,200 square kilometers. Since 1949, over 55,000 cases have been registered, with Taixing accounting for the highest number of patients. The proportion of new cases with MB and G2D was higher compared to other regions. As a result, Jiangsu has been considered a priority area for public health interventions in China.

**Methods:**

This paper mainly described the population, time, and spatial distribution of the newly detected leprosy cases in Jiangsu Province between 2005 and 2020. In this study, all the data were entered into Microsoft Excel and SPSS for the descriptive analysis. ArcGIS was applied to create statistical maps, and Geoda was used to conduct spatial autocorrelation analysis with local Moran's *I* statistics (LISA). The epidemiological data were obtained from LEPMIS. In addition, population data were obtained from the Statistical Yearbook of Jiangsu Province.

**Results:**

During the study period, 363 new cases were reported. Of these, 232 were men and 131 were women (1.77:1). The mean age at diagnosis was 60.56 years, and no adolescent cases were identified. Three hundred and twenty-seven (90.08%) were diagnosed with MB and 36 (9.92%) with PB. 31.68% (115/363) of the patients presented with G2D. Farmers accounted for 74.9%, and most cases were identified in skin clinics (248, 68.32%). We observed a decreasing trend in detection rate, with a higher concentration of new cases diagnosed between July and October. Spatial analysis showed that the new cases were primarily distributed in the northwest of Jiangsu province, and Suqian has the highest incidence of leprosy. Special attention should be paid to Wuzhong, a county with a potential risk of inter-provincial transmission. Furthermore, 55 new cases came from other Chinese provinces but lived in Jiangsu.

**Conclusion:**

The NCDR of leprosy decreased, but the new cases showed disabilities, a sign of the late diagnosis. The results indicated that some regions were still suffering from the burden of leprosy. Thus, we recommend that the government should adopt effective strategies to promote leprosy control. The main priorities for eliminating new cases were to provide sustainable financial support, improve the quality of clinical services, strengthen preventive intervention and rehabilitation services for disabilities, provide health education among high-risk populations, and explore new approaches.

## Introduction

Leprosy is caused by Mycobacterium leprae or Mycobacterium lepromatosis, which has a long incubation period (2–12 years). In 1873, Mycobacterium leprae was discovered in Bergen (Norway) by the biologist Hansen (1). After 135 years, Mycobacterium lepromatosis was firstly isolated from the skin tissue of two patients in 2008 (2). The cases presented with the special multibacillary form of leprosy, which we can call diffuse lepromatous leprosy (DLL). Dual infection with Mycobacterium lepromatosis and Mycobacterium leprae has also been reported in Mexico and Singapore (two cases) (3, 4). After that, more and more patients were discovered. In a study conducted by Deps (5), more than 150 cases were reported worldwide, mainly from three endemic countries (Brazil, Philippines, and Myanmar) and three non-endemic countries (Mexico, Malaysia, and the USA). Notably, *M. lepromatosis* has also been reported in Colombia (6, 7). For instance, five patients tested positive for *M. lepromatosis* during 2006–2016, and the results suggest that this pathogen was present when this mycobacterium was first reported (2008). However, it has never been reported in China before, which greatly hinders the study of the species (8). In addition, several reports suggest that animals can also be infected with Mycobacterium lepromatosis (red squirrels, armadillos, etc.) (9, 10).

The transmission of leprosy remained unknown, although it could be transmitted person-to-person by nasal droplets (11). Delayed diagnosis could cause irreversible peripheral nerve damage and disability. As recommended by the World Health Organization (WHO), early diagnosis and multidrug therapy (MDT) remain key strategies for leprosy control (12). WHO has adopted the elimination of leprosy as a public health goal to reduce the global incidence to <1/100,000 by 2000, and most countries have already achieved the goal (13). However, there were 208,641 new cases worldwide in 2018, with 192,713 new patients being treated. Studies demonstrated that leprosy is prevalent in tropical countries, especially in developing countries, such as India, Brazil, and Indonesia (14–18). In India, leprosy was eliminated until 2005, and new cases reported remained almost constant over the past decades. The National Leprosy Elimination Programme (NLEP) has implemented new initiatives and interventions to accelerate progress toward the goal of elimination. From 2016 to 2018, the number of newly detected cases in India declined significantly by ~15,000. Grade 2 disability (G2D) cases decreased from 5,245 to 3,666, and new pediatric cases dropped to <10,000 (19). Brazil is also one of the countries with the highest number of cases. The government has taken extensive public health measures to prevent disability recently. However, the data indicated that more than 2,000 patients had been diagnosed with leprosy-related G2D annually (20). Vieira et al. also found that the proportion of children (<15 years) presenting with G2D remained high (21). In Indonesia, leprosy control programs have been scaled back due to the loss of resources and clinical expertise. The incidence remained stable (>6/100,000), and 15% of Indonesian adolescent patients presented with G2D (22).

In the last century, there were more than 500,000 leprosy patients in China, mainly concentrated in Yunnan, Guizhou, Sichuan, and Jiangsu Province (23–26). According to the WHO definition (<1/100,000), China eliminated leprosy nationwide in 1981 (27). However, there were still 300–500 new cases of leprosy annually. The spread continues, and it has remained a major public health problem. The Chinese Ministry of Health initiated the National Leprosy Control Plan (2011–2020), which aims to control the incidence and its hazards through direct public health investments (28). China's *Leprosy Management Information System* (LEPMIS) was an upgraded version of the former national leprosy recording and reporting system. According to LEPMIS, the incidence rate of leprosy declined significantly from 2011 to 2020, and the detection rate was 0.032/100,000 residents at the national level in 2020 (29). However, in 2018, the newly detected G2D cases rate was 19.0%, which was higher than the general level (30).

Jiangsu Province is located in the Yangtze River Delta region with a total area of 107,200 square kilometers. The province is divided into 13 municipalities and 95 counties/districts. Since 1949, over 55,000 cases have been registered, and Taixing county registered the highest amount and incidence rate of leprosy patients in China. In Jiangsu province, the highest average detection rate was 8.69 per 100,000 in 1955–1959, while the highest 5-year average prevalence rate was 60.36 per 100,000 in 1970–1975. After years of effort, the number of patients has decreased significantly. From a historical point of view in China, the incidence of leprosy was much higher in coastal regions and the Yangtze River valley. Meanwhile, the province's subtropical monsoon climate predisposes it to the spread of leprosy (21). Thus, Jiangsu has been considered a priority region for public health interventions in China.

Recently, WHO announced its new plan: Toward zero leprosy-Global Leprosy (Hansen's disease) strategy 2021–2030. The key objectives of the plan were as follows. Firstly, to achieve zero new cases of indigenous leprosy worldwide. Secondly, new patients were reduced to 65,000 per year; Thirdly, the G2D rate decreased to 0.12 per million. At last, the prevalence of adolescents dropped to 0.77 per million (31). The plan indicated that there was still a long way to go before eradication. In recent years, geographic information systems (GIS) have been widely used in public health, especially epidemiology. Therefore, in this paper, we mainly described the epidemiological characteristics of leprosy in Jiangsu Province during 2005–2020 with Spatio-temporal and descriptive analysis, aiming to provide epidemiological clues for developing eradication strategies.

## Methods

### Ethical announcement

Ethical approval is not required to analyze LEPMIS data in common public health practice. Data processing and analysis strictly respected all the patients' confidentiality.

### Study area and population

Jiangsu Province is located in the Yangtze River Delta urban agglomeration on the eastern coast of China and covers an area of 107,200 square kilometers. It is situated at longitude 116°21′ to 121°54′E and latitude 30°46′ to 35°08′N, with the Yellow Sea to the east, Zhejiang/Shanghai to the south, Anhui Province to the west, and Shandong Province to the north. The province is divided into 13 municipalities and 95 counties/districts. By 2020, the resident population of Jiangsu Province has surpassed 8.45 million, and its GDP ranks first in China.

### Classification and diagnosis

The diagnosis of leprosy was based on the “*Leprosy Diagnostic Standards WS291-2018*” and the “*Leprosy Prevention Manual for Primary Care Practitioners (PCPS)*” (32). The newly detected cases were cases diagnosed with leprosy including that moved from other provinces to Jiangsu after diagnosis in that year. A two-group classification system developed by the WHO was used to classify patients as multibacillary (MB) or paucibacillary (PB) (31). Disability was classified into three levels (0, 1, 2) according to WHO (Grade 0 disability: No loss of sensation, no visible deformities, and no eye problems associated with leprosy. Grade 1 disability: leprosy-related sensory loss or eye problems. Grade 2 disability: Visible deformity or severe visual impairment.) (33).

### Data collection

Epidemiological data in Jiangsu were obtained from China's *Leprosy Management Information System* (LEPMIS). LEPMIS includes basic information on newly detected cases: Demographic information (Gender, education, occupation, date of birth, ethnicity, and geographical information) and clinical information (Date of diagnosis, age at the confirmed date, date of symptom onset, time from symptom onset to diagnosis, grade of physical impairment, detection method.). The Chinese government developed LEPMIS in 2010, which reports data not only on newly diagnosed patients and the patients undergoing treatment but also on the patients who have been cured and those who have achieved lifelong management. New Case Detection Rate (NCDR) was defined as the number of new cases detected annually per 100 million of the general population (34). The average interval between patient delay from the onset of the disease and diagnosis was calculated in months. In addition, all the population data were obtained from the Statistical Yearbook of Jiangsu Province (http://tj.jiangsu.gov.cn/2021/nj03/nj0301.htm).

### Statistical analysis

All the data were entered into Microsoft Excel (Version 2021, Microsoft Corporation, Redmond, WA, USA) and SPSS (version 20.4, IBM Corporation, Armonk, NY, USA) for the descriptive analysis. Qualitative variables were presented as frequencies and percentages. The charts used in the paper were drawn by Original (Version 2021, Microsoft Corp, Redmond, WA, USA). ArcGIS (version 10.6, ESRI Inc., Redlands, CA, USA) was applied to create statistical maps at the city/county level, and Geoda (version 1.8.61, University of Chicago, IL, USA) was used to conduct spatial autocorrelation analysis. Spatial autocorrelation is defined as the correlation of a variable in adjacent spatial locations, which is focused on a metric spatial unit attribute value. It is aimed to discover the spatial distribution characteristics of a particular region. Local Moran's *I* is a measure of spatial autocorrelation across the study area (28). It ranges from −1 to +1, where 0 indicates no spatial auto-correlation, and the values −1 and +1 indicate negative and positive auto-correlation, respectively. In this study, we explored the aggregation region by space autocorrelation analysis. The different colors on the spatial autocorrelation map represent the types of spatial autocorrelation between different regions (High-High, High-Low, Low-Low, and Low-High).

## Results

### Demographic features of newly detected cases in Jiangsu province

**Figure 1:**
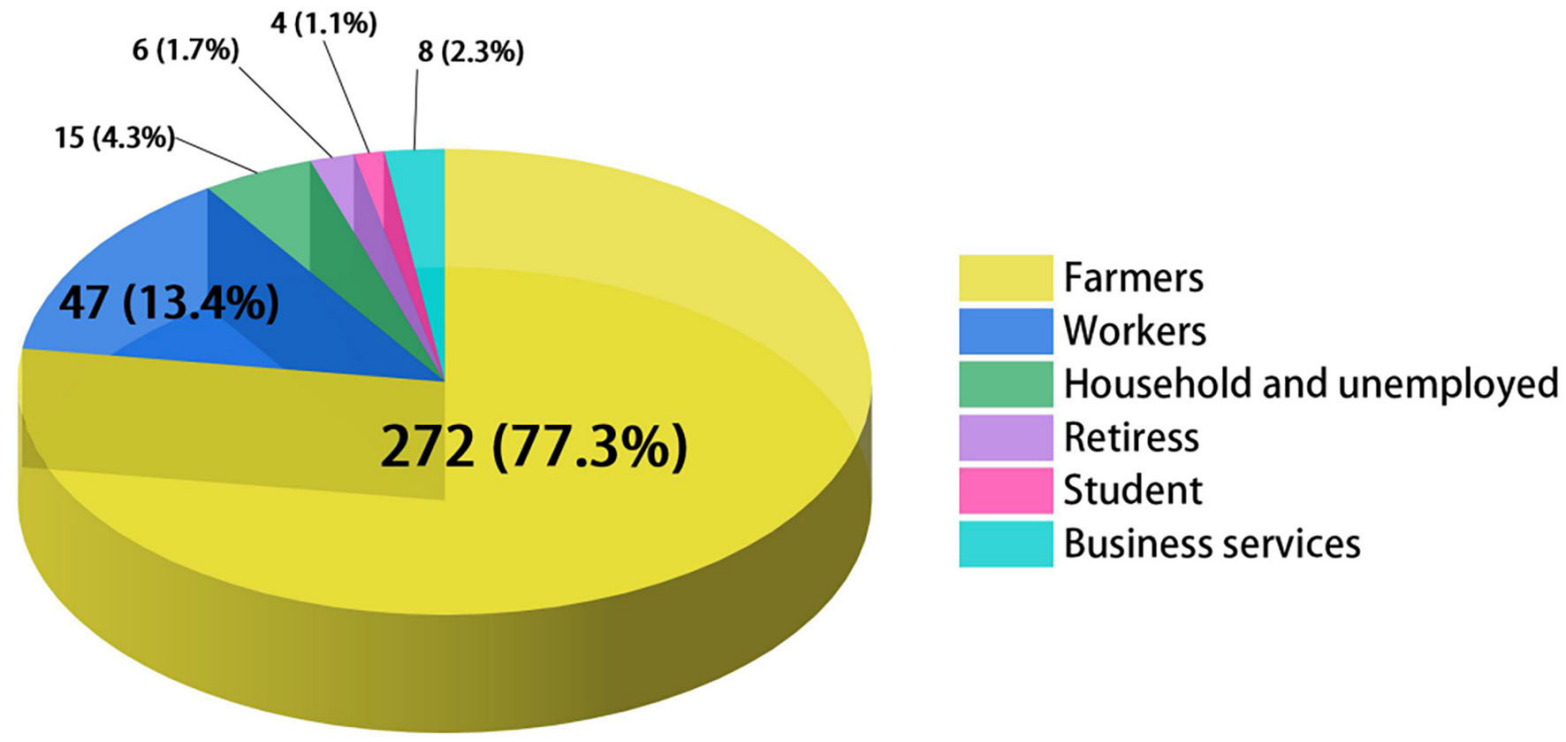
Occupational distribution among newly detected leprosy patients in Jiangsu, 2005–2020. Different colors represent different occupations.

As it was shown in Table 1, 363 newly detected patients were reported in Jiangsu Province during the study period. Among these cases, 232 were male, and 131 were female, with a ratio of 1.77:1. The largest ratio of new cases of leprosy between men and women was recorded in 2017 (6.50:1). It can be seen that the population of Jiangsu Province has continued to grow in recent years, while the annual detection rate of leprosy had decreased from 36/100,000,000 to 7/100,000,000 between 2005 and 2020. The average NCDR was 28 per 100,000,000 population. The results indicated an aging trend of newly diagnosed cases, and the mean age at diagnosis was 60.56 years. No adolescent cases were identified during the period. In addition, the occupational distribution was farmers (272, 74.93%), workers (47, 12.95%), household and unemployed (15, 4.13%), retirees (6, 1.65%), students (4, 1.10%), business services (8, 2.20%), and others (11, 3.03%) (Figure 1).

**Table 1:** The demographic data of newly detected leprosy cases in Jiangsu, 2005–2020.

**Year**	**Total population in Jiangsu province (× 100,000)**	**NCDR (/100,000,000)**	**Cases of male (%)**	**Cases of female (*n*/%)**	**Sex ratio**	**Average age, (SD)**	**No. of new cases (*n*/%)**
2005	758.8	36	17 (62.96)	10 (37.04)	1.70	59.85 (13.95)	27 (7.44)
2006	765.6	33	16 (64.00)	9 (36.00)	1.78	64.20 (16.74)	25 (6.89)
2007	772.3	44	21 (61.76)	13 (38.24)	1.62	62.26 (16.58)	34 (9.37)
2008	776.3	39	18 (60.00)	12 (40.00)	1.50	63.83 (13.94)	30 (8.26)
2009	781.0	50	24 (61.54)	15 (38.46)	1.60	63.61 (16.55)	39 (10.74)
2010	786.9	46	23 (63.89)	13 (36.11)	1.77	61.56 (14.88)	36 (9.92)
2011	802.3	34	18 (66.67)	9 (33.33)	2.00	59.59 (14.33)	27 (7.44)
2012	812	38	19 (61.29)	12 (38.71)	1.58	64.35 (14.97)	31 (8.54)
2013	819.2	26	15 (71.43)	6 (28.57)	2.50	58.76 (20.14)	21 (5.79)
2014	828.1	34	17 (60.71)	11 (39.29)	1.54	54.71 (16.29)	28 (7.71)
2015	831.5	16	8 (61.54)	5 (38.46)	1.60	55.31 (18.19)	13 (3.58)
2016	838.1	17	9 (64.29)	5 (35.71)	1.80	53.07 (20.42)	14 (3.86)
2017	842.3	18	13 (86.67)	2 (13.33)	6.50	59.80 (14.24)	15 (4.13)
2018	844.6	12	6 (60.00)	4 (40.00)	1.50	49.70 (19.36)	10 (2.75)
2019	846.9	8	4 (57.14)	3 (42.86)	1.33	62.28 (16.04)	7 (1.93)
2020	847.5	7	4 (66.67)	2 (33.33)	2.00	62.00 (16.12)	6 (1.65)
Total	–	28	232 (63.91)	131 (36.09)	1.77	60.56 (16.24)	363 (100.00)

NCDR, new case detection rate; SD, standard deviation; sex ratio: male/female.

### The comparison of new cases in Jiangsu province with three endemic provinces of China

**Figure 2:**
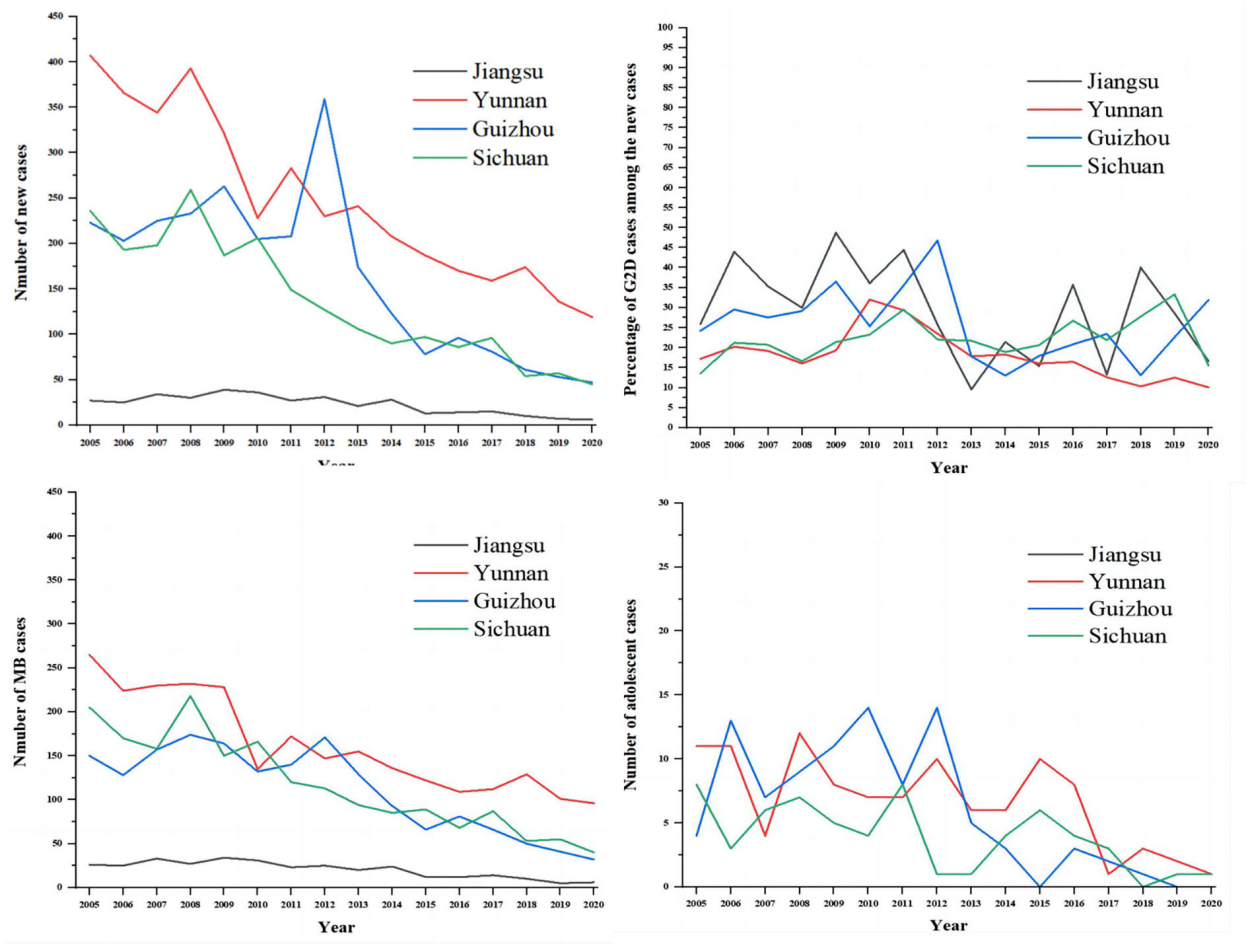
The comparison of the epidemiological features of new cases between Jiangsu, Yunan, Guizhou, and Sichuan. The gray line represents Jiangsu province, the red line represents Yunnan province, the blue line represents Guizhou province, and the green line represents Sichuan province.

As shown in Figure 2, we compared the situations of leprosy (New cases, G2D, MB, and adolescents) in Jiangsu Province with the other three endemic regions of China (Yunnan, Guizhou, and Sichuan). The results showed that the epidemiological characteristics of leprosy in Yunnan, Guizhou, and Sichuan were similar to Jiangsu province. It also can be seen that the number of new cases and MB patients were higher in Yunnan annually than in other regions, followed by Guizhou and Sichuan. Meanwhile, Guizhou province had the highest number of these indicators in 2012 (359, 171, 168, and 14 cases, respectively). Despite the decline of new cases in Jiangsu, the proportion of G2D patients among new cases per year was the highest among the four provinces. In addition, sporadic cases of adolescents (<15) were reported annually in Yunnan, Guizhou, and Sichuan during the study period (2005–2020).

### Epidemiological characteristics of the newly detected cases in Jiangsu province

**Figure 3:**
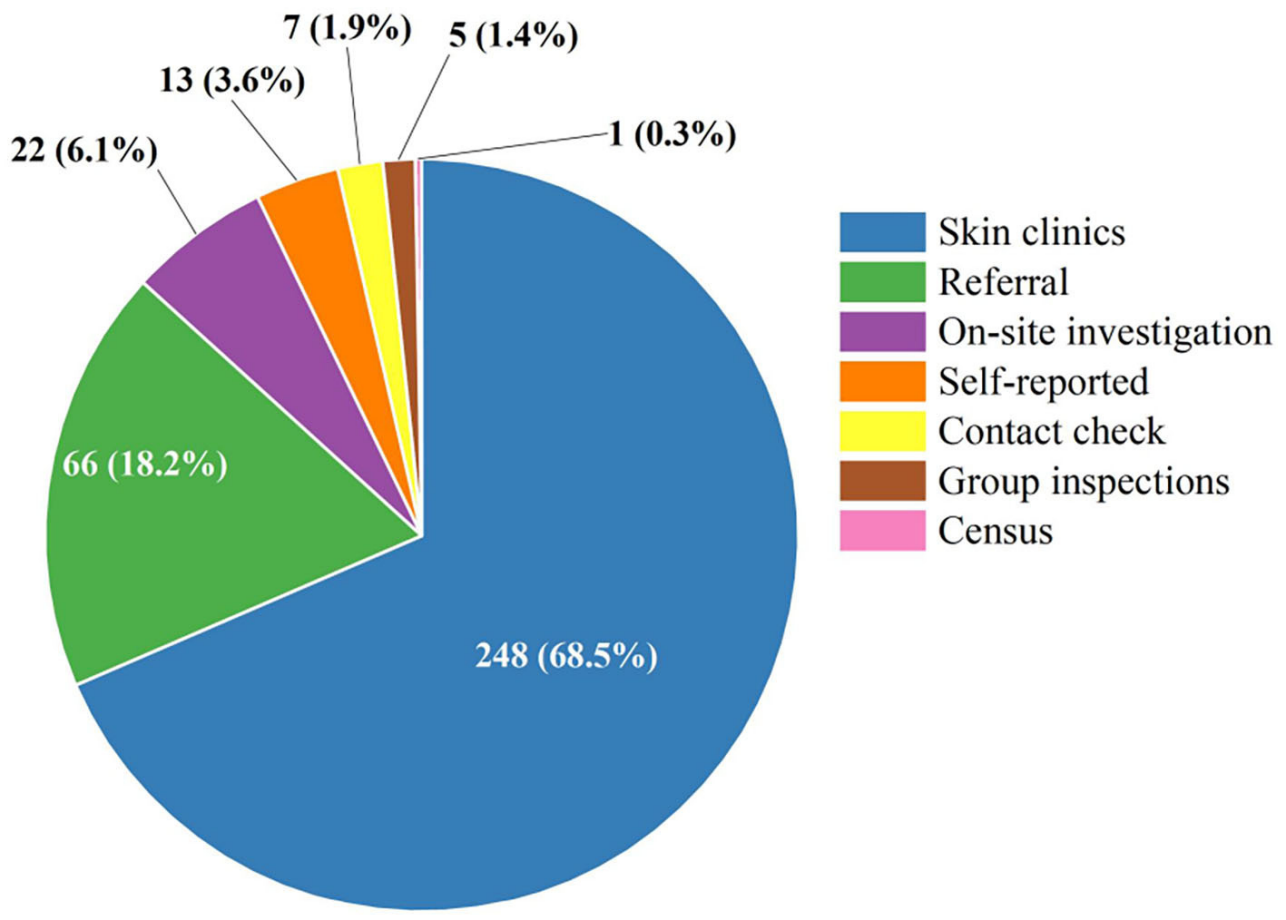
The distribution of the detection mode among the newly detected leprosy cases in Jiangsu Province, 2005–2020. Different colors represent different ways of discovery. Referral: referral from other departments of the general hospital.

Table 2 shows the epidemiological features of new cases. In total, 327 (90.08%) newly detected leprosy cases were diagnosed with MB and 36 (9.92%) with PB in the province. The number of MB cases declined annually from 26 in 2005 to 6 in 2020, while the ratio of MB cases remained high and stable in the study period. The mean time to diagnosis was 44.68 months, with the longest mean time to detection in 2009 (88.38 months). Among all the new cases, 31.68% (115/363) showed disability of G2D, 21.21% (77/363) showed G1D, and the remaining new cases (171/363) showed no visible disabilities (Figure 2). The annual G2D rate decreased from 25.93 to 16.67%. In 2009, the rate of G2D was the highest (48.72%). As it can be seen in Figure 3, most new cases were detected in the skin clinic (248, 68.32%), followed by “referral from other departments of the general hospital” (66, 18.18%), “field investigation” (22, 6.06%), “self-report” (13, 3.58%), “contact examination” (7, 1.93%), “group examination” (5, 1.38%), and “census” (1, 0.28%).

**Table 2:** The epidemiological data of newly detected leprosy cases in Jiangsu province, 2005–2020.

**Year**	**MB (*n*/%)**	**PB (*n*/%)**	**Average delay time (months)**	**G2D (*n*/%)**	**No. of cases identified in skin clinics (*n*/%)**	**No. of new cases (*n*/%)**
2005	26 (96.30)	1 (3.70)	23.44	7 (25.93)	22 (81.48)	27 (7.44)
2006	25 (100.00)	0 (0.00)	25.64	11 (44.00)	20 (80.00)	25 (6.89)
2007	33 (97.06)	1 (2.94)	64.68	12 (35.29)	25 (73.53)	34 (9.37)
2008	27 (90.00)	3 (10.00)	33.13	9 (30.00)	26 (86.67)	30 (8.26)
2009	34 (87.18)	5 (12.82)	88.38	19 (48.72)	25 (64.10)	39 (10.74)
2010	31 (86.11)	5 (13.89)	54.19	13 (36.11)	25 (69.44)	36 (9.92)
2011	23 (85.19)	4 (14.81)	41.56	12 (44.44)	17 (62.96)	27 (7.44)
2012	25 (80.65)	6 (19.35)	29.42	8 (25.81)	19 (61.29)	31 (8.54)
2013	20 (95.24)	1 (4.76)	27.81	2 (9.52)	14 (66.67)	21 (5.79)
2014	24 (85.71)	4 (14.29)	34.29	6 (21.43)	16 (57.14)	28 (7.71)
2015	12 (92.31)	1 (7.69)	23.62	2 (15.38)	11 (84.62)	13 (3.58)
2016	12 (85.71)	2 (14.29)	37.21	5 (35.71)	10 (71.43)	14 (3.86)
2017	14 (93.33)	1 (6.67)	45.53	2 (13.33)	8 (53.33)	15 (4.13)
2018	10 (100.00)	0 (0.00)	79.60	4 (40.00)	3 (30.00)	10 (2.75)
2019	5 (71.43)	2 (28.57)	45.29	2 (28.57)	5 (71.43)	7 (1.93)
2020	6 (100.00)	0 (0.00)	25.33	1 (16.67)	2 (33.33)	6 (1.65)
Total	327 (90.08)	36 (11.01)	44.68	115 (31.68)	248 (68.32)	363 (100.00)

G2D, grade 2 disability; MB, multibacillary; PB, paucibacillary.

### Temporal distribution of the newly detected cases

**Figure 4:**
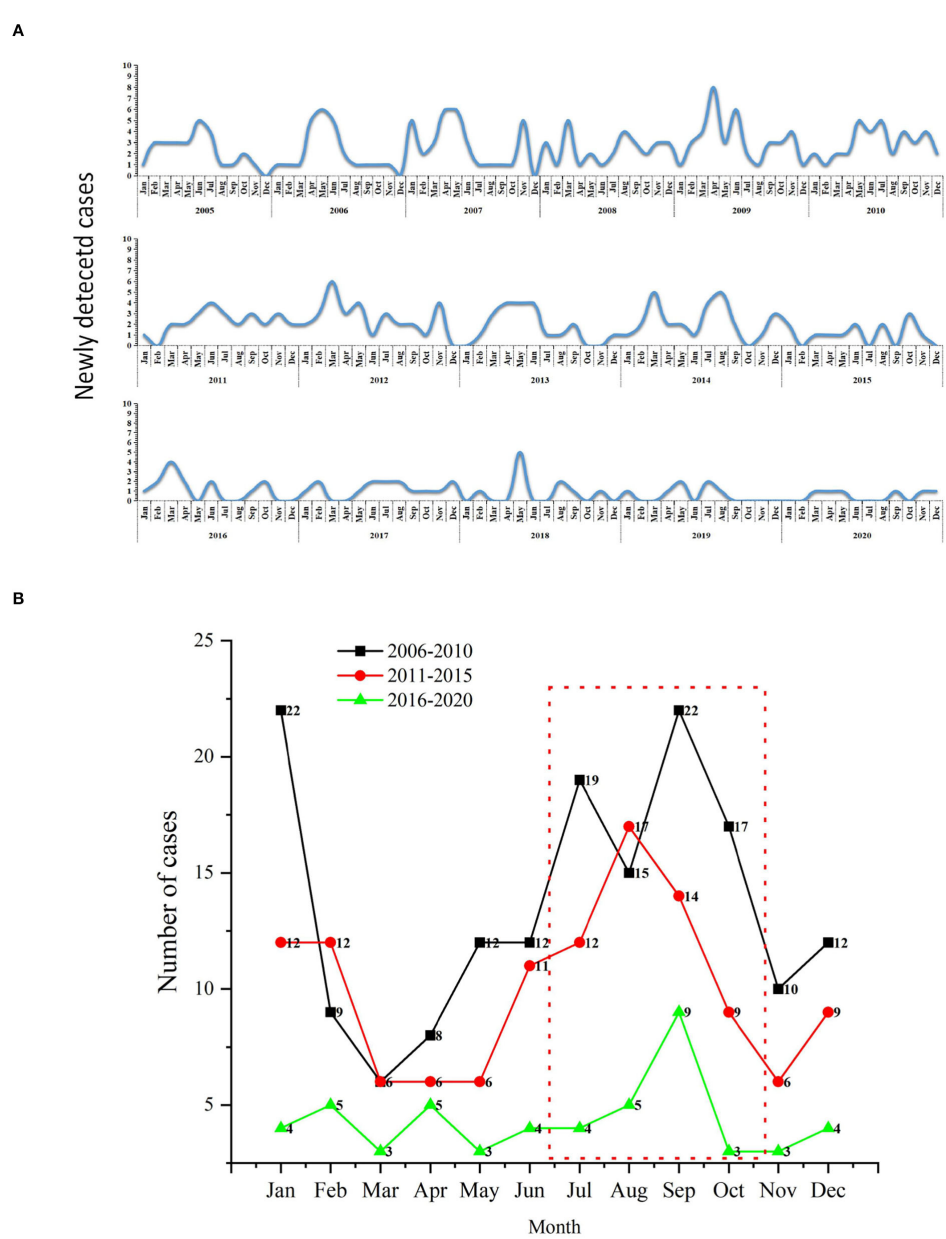
The temporal distribution of newly detected leprosy cases in Jiangsu Province, 2005–2020. **(A)** Monthly detection rate of the new cases; **(B)** Comparison of monthly detection rates per 5 years.

Figures 4A, B was shown the distribution of new cases at the city and preferccture level city (2005–2020). There was a long-term downward trend of newly discovered leprosy cases in Jiangsu province, ranging from 26 to 6, with the lowest number of cases detected in 2009 (34 cases). However, there was an increasing trend from 2005 to 2009 (27 cases to 39 cases). The results showed a higher concentration of new cases diagnosed between July and October. September 2009 was the month with the highest number of new cases detected (eight cases). And then, we divided the study period into three parts (2006–2010, 2011–2015, and 2016–2020). The highest incidence was observed in 2006–2010, while the lowest was observed in 2016–2020 (Figure 4B). Furthermore, the lowest detection rate was observed in March.

### Spatial analysis of new cases by GIS

**Figure 5:**
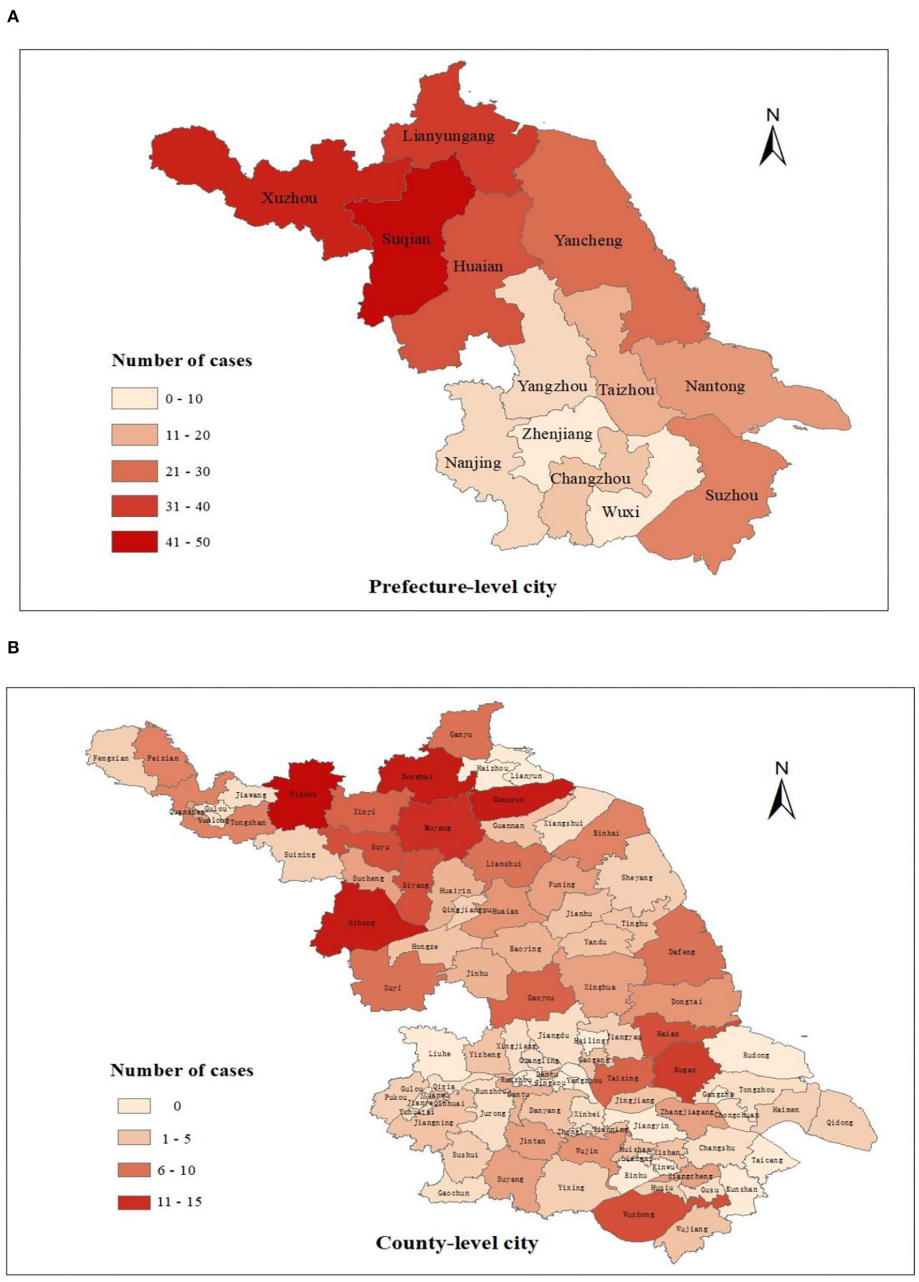
The geographical distribution of newly detected cases in Jiangsu Province, 2005–2020. **(A)** Spatial distribution of new cases at the prefecture level; **(B)** Spatial distribution of new cases at the county level.

**Figure 6:**
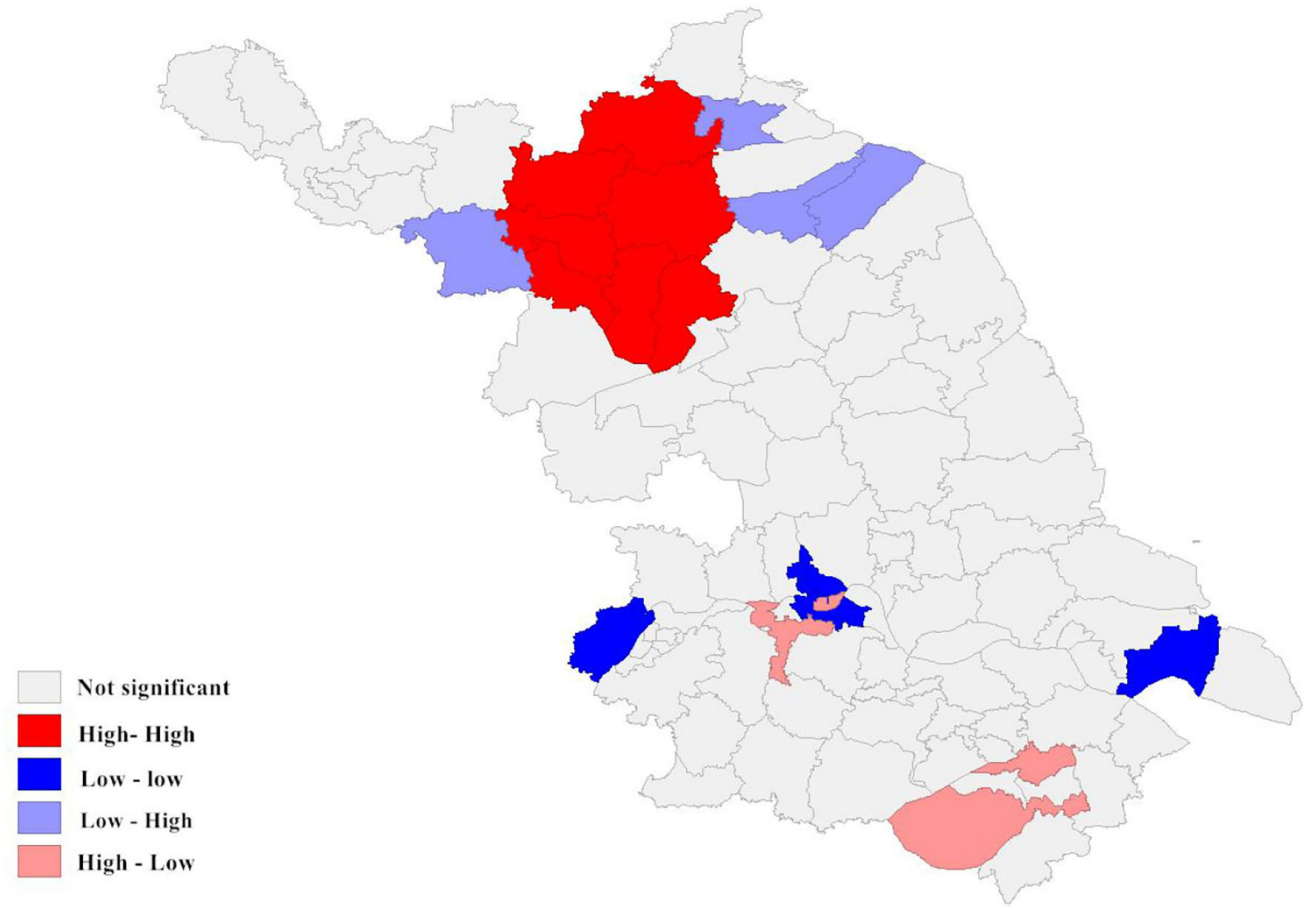
Local spatial autocorrelation analysis of newly detected leprosy cases in Jiangsu Province from 2005 to 2020. The different colors on the spatial autocorrelation map represent the types of spatial autocorrelation between different regions. For example, the low-low cluster indicates that the area is a low-incidence area, and the surrounding areas should also be low-incidence areas.

The spatial distribution of new cases at the prefecture and county level city is shown in Figures 5, 6. As the results indicated, newly detected leprosy cases have been reported among 81 counties of 13 prefecture-level cities in Jiangsu Province. The amount of these cases in the northern area (Xuzhou, Suqian, Xuzhou, etc.) was relatively higher than in the southern region (Wuxi, Zhenjiang, etc.) (Figure 5). At the prefecture-level city level (Figure 6), Zhenjiang (eight cases) and Wuxi had the lowest number of new cases (nine cases). In comparison, Suqian (50 cases) had the highest number of new cases. At the county level, ten counties had more than ten newly detected cases, followed by Wuzhong (10 cases), Huaian (10 cases), Suyu (10 cases), Siyang (10 cases), Rugao (11 cases), Muyang (13 cases), Donghai (14 cases), Guanyun (14 cases), Sihong (14 cases), Pizhou (15 cases).

Out of the 363 cases, 308 (84.85%) were born in Jiangsu province, and 55 were from other domestic provinces in China but settled in Jiangsu province, ranging from 1 to 14 (Table 3). These cases were mainly from Guizhou (14 cases) and Sichuan province (10 cases), which are regarded as highly endemic provinces in southwest China (23).

**Table 3:** Distribution of 55 newly detected leprosy cases according to registered provinces, 2005–2020.

**Province**	**Male (*n*/%)**	**Female (*n*/%)**	**Total (Assistive Technology *n*/%)**
Anhui	3 (42.86)	4 (57.14)	7 (12.73)
Gansu	1 (100.00)	0 (0.00)	1 (1.81)
Guizhou	8 (57.14)	6 (42.86)	14 (25.45)
Hebei	1 (100.00)	0 (0.00)	1 (1.81)
Henan	1 (33.33)	2 (66.67)	3 (5.45)
Heilongjiang	0 (0.00)	1 (100.00)	1 (1.81)
Hubei	2 (66.67)	1 (33.33)	3 (5.45)
Hunan	0 (0.00)	2 (100)	2 (3.64)
Jiangxi	3 (75.00)	1 (25.00)	4 (7.27)
Shanxi	0 (0.00)	2 (100.00)	2 (3.64)
Sichuan	7 (70.00)	3 (30.00)	10 (18.18)
Yunnan	1 (25.00)	3 (75.00)	4 (7.27)
Chongqing	2 (66.67)	1 (33.33)	3 (5.45)
Total	29 (52.73)	26 (47.27)	55 (100.00)

According to the local autocorrelation Index (LISA) analysis, there was a significant spatial autocorrelation (Moran's index = 0.970) in the incidence of newly detected leprosy cases within the study area between 2005 and 2020. As the results indicated, the High-High region was mainly concentrated in the northwest of Jiangsu Province and comprised seven county-level cities, mainly including Suqian. The Low-Low cluster was distributed in the south, such as the Gulou District in Nanjing. It can be seen that Wuzhong was the main region of the High-low cluster. Although the incidence of leprosy in Wuzhong was not high, it borders a high prevalence area of leprosy in Zhejiang. The phenomenon indicated that there was a potential risk of inter-provincial transmission. In addition, three county-level cities formed the Low-High cluster (Figure 4).

## Discussion

Up to now, leprosy is far from being eradicated, as more than 200,000 new cases are detected each year globally (15). Although China is not classified as a priority country for leprosy intervention by WHO, there are still 300–400 new cases annually, which continues to pose challenges to leprosy prevention and control (25). In fact, we still lack an effective vaccine and reliable diagnostic tests to protect the population at risk (11). Therefore, how to pick up the few scattered leprosy cases latent in a large population poses a threat to China and the entire world. Even in the post-elimination era, efforts are still needed to reduce the disease burden and to maintain the necessary control activities.

In this study, we found that there were 363 new cases of leprosy were reported during 2005–2021. Of these, 232 were men, and 131 were women (1.77:1). Similar results have also been found in other countries (India, Bangladesh, and Brazil) (15, 35–38). Although no medical evidence suggests that men are more likely to develop leprosy than women, the reason may be that men have more opportunities to be exposed to a more intensive social network (39). The inconsistency between the operating hours of health care facilities and the workday, the belief that men are less sensitive to leprosy compared to women, and the barriers associated with difficulties in accessing health care services may also contribute to the increased burden of leprosy among the male population (38). Farmers accounted for 74.9%, consistent with previous studies (23, 40). The result indirectly proves the low level of education of the new cases in Jiangsu province and their ignorance of the disease status as well as the modes of transmission. Occupation with high environmental exposure (e.g., agriculture), low education level, and poverty were both risk factors for leprosy, which might explain the high proportion of farmer patients in this paper (41). Fear, stigma and, ignorance of leprosy persist among farmer patients.

Zero leprosy means 0 infections, 0 new cases, 0 disability, 0 stigmas, and 0 discrimination (42). However, funders, health planners, and populations seem to equate “eliminating leprosy as a public health problem” (prevalence <1/100,000) with eradicating leprosy, i.e., “reducing NCDR to zero in a defined geographic area”. To be honest, elimination efforts aim to control the disease burden rather than infection. Patients undergoing treatment or who have completed MDT still suffer from deep-rooted social discrimination, which burdens society and families heavily (43). In other aspects, misdiagnosis can increase the financial burden until the patient is properly diagnosed. Going to the local leprosarium each month also consumed more time and money to pick up medication after diagnosis. In a survey conducted in Guangzhou, the median annual cost of leprosy to residents was $152.4, with $309.7 after diagnosis and $91.9 for complications of leprosy, indicating that the burden remained high from the data results (44).

Experts consider that it is difficult to achieve eradication before 2030 in Jiangsu. It is the unique nature of Mycobacterium leprae and its interaction with the host immune system that makes this bacterium unculturable (45). The fact has severely hampered the development of effective vaccines and reliable diagnostic tests. It is also the reason that there have been sporadic cases of leprosy in recent years. Another typical barrier to eliminate leprosy is the deep-rooted discrimination against leprosy patients in society and the stigma that patients suffered. Stigma contributes to a variety of discrimination that ultimately denies individuals or communities social acceptance, reduces individual opportunities, and ultimately increases social inequality (46). The discrimination and fear of patients in society are difficult to change in a short period of time and represent an important factor in determining whether a patient can return to society. In particular, the isolation policy implemented in Jiangsu Province for leprosy patients has led to more serious discrimination against patients. Experts have pointed out that stigma is a significant barrier to participation in care, health-seeking behaviors, and adherence to treatment for a range of health conditions (47).

In Jiangsu province, leprosy control is a purely governmental plan implemented at the national level. The provision of appropriate rehabilitation programs for patients with disabilities and the control of current sources of infection/transmission was inadequate, and more patients with deformities need appropriate treatment and care. Instead, the focus of studies in many countries has shifted away from ensuring adequate resources to enhance the results achieved to date. According to LEMPIS, more than 10,000 patients in Jiangsu Province still have varying degrees of disability after treatment. The government has conducted relatively homogeneous studies on the health hazards associated with the stigma of deformities, such as anxiety or depression (32). This trend encourages isolated research on leprosy-related stigma and stifles innovative public health responses. Furthermore, funding for leprosy planning was drastically reduced after the announcement of the elimination of leprosy. Consequently, compared to other provinces in China, Jiangsu Province has recently borne a heavy burden of leprosy (31). Therefore, we compared the situation of leprosy in Jiangsu (New cases, G2D, MB, and adolescents) with other endemic regions of China (Sichuan, Yunan, and Guizhou). As the results indicated, all the provinces showed a downward trend in the number of new cases. The highest incidence was in Yunnan, followed by Guizhou and Sichuan. Notably, despite the low prevalence, Jiangsu province has the highest proportion of patients with G2D.

NCDR, delayed diagnosis, adolescent cases, and G2D rate are the most critical indicators to evaluate the effectiveness of leprosy prevention and control (48). These indicators provide information about other factors that indirectly influence case detection, such as medical personnel's ability to recognize early symptoms. As the results indicated, the NCDR of leprosy has dropped from 36/100,000,000 to 7/100,000,000. In 2018, the rate in Jiangsu (12/100,000,000) was much lower than that of China (37/100,000,000) and the global average (2740/100,000,000) (49). Similar trends of new cases were also reported in other regions (50, 51). For instance, the number of patients registered in Korea fell from 4,393 to 166 during 1977–2017, while new cases fell from 39 to 4 from 1996 to 2017. However, there was an increasing trend in the detection rate of leprosy during 2005–2009 in Jiangsu Province. The trend was mainly due to the relaxation of leprosy control and the appearance of drug resistance (dapsone, rifampin, and ofloxacin), which has led to an increase in new cases (31, 52). As a result, the government took adequate measures to control the disease, and the epidemic was brought under control. The highest detection rate was in 2006–2010, while the lowest was recorded in 2016–2020. The achievements were primarily attributed to the National Leprosy Control Plan (2011–2020), launched by the Jiangsu Provincial Health Planning Commission, with the aim of controlling leprosy and its hazards through direct public health investments (8). Furthermore, we observed a higher concentration of new cases detected between July and October, while the lowest was in March. Although leprosy is not traditionally considered a seasonal disease, studies have considered the highest positivity rate for Mycobacterium leprae in the nasal mucosa during the monsoon season (39, 53). Our results may support the idea. The seasonal pattern of leprosy might be considered when planning control actions or expanding the provision of medical services.

**The NCDR of leprosy decreased, but the new cases showed disabilities, a sign of late diagnosis**. Preventing disability is one of the priorities of the Global Leprosy Strategy. The rates increase with age, and the stigma associated with disability severely affects patients' physical and mental health, especially in adolescents (54). Physical disability is irreversible and leads to emotional, social, and economic damage to the patients. We strongly recommend that patients be followed up during and after treatment, besides monitoring the progression of physical disability. G2D mainly represents the capacity of the health system to identify and treat leprosy at an early stage, as well as the level of public awareness of the early symptoms. The proportion of G2D cases among new cases in Jiangsu remains high in Jiangsu (31.68%), and it is significantly higher than in other provinces. We can conclude that the disability of leprosy remains a major public health problem in Jiangsu. It is important to review the chemoprophylaxis offered to patients, and effective measures should be taken prospectively to prevent disability. For instance, improving leprosy surveillance system capabilities for early detection could help reduce the proportion of G2D, especially in low-endemic areas (55–57). Specialized health education activities must be conducted for the general public to eliminate public misconceptions and prejudices about leprosy and promote self-reporting and early detection. In addition, most new cases were detected as MB patients (90.08%). The high proportion of MB cases also indirectly indicates a delay in diagnosis (23). Compared to PB patients, MB patients have more severe symptoms, more complications, a higher disability rate, and require a longer course of MDT (58). In summary, all the results demonstrate a serious late diagnosis and underdiagnosis of new cases in Jiangsu Province, and the chain of transmission still exists (38).

Early diagnosis of leprosy is essential to reduce bacterial reservoirs and the risk of malformations. However, the mean time to diagnosis for new cases was 44.68 months in Jiangsu Province, which is significantly higher than in other provinces in China or even the world (23–25). It is more than the average delay of 37 months reported in Purulia (59). In contrast, the average delay for patients was 7.9 months in a tertiary care hospital in South India (60). Srinivas et al. found that the probability of disability was 1.6 times higher if patients were diagnosed with a delay time of more than 3 months (61). Barriers to early detection with responsive symptoms included a lack of awareness of the complications associated with the tropical disease and regarding health as a low priority compared to wage earning (62). Furthermore, in the early stages of leprosy, patients often face misdiagnosis because the various early symptoms can be non-specific, which makes early diagnosis extremely difficult. The typical clinical symptoms include discoloration, nodules, and deformities or disfigurement (63). A similar conclusion has also been obtained by Chu et al. (57) in Shandong Province. Shandong province has many worthwhile experiences in improving the detection rate of leprosy. For example, the government will award $5,000 ($696) for each case with a delayed diagnosis of <12 months and no apparent disability.

Longer diagnosis times and fewer pediatric cases have led to an increasing age range for new patients. Compared to adults, children do not have a mature immune system, and the disease is hereditary, so active monitoring of pediatric cases is critical. In a retrospective study conducted in Cuba, more than half of the children with leprosy had a definite source of infection, and the vast majority of these were their grandparents (64). It is worth mentioning that special attention to this epidemiological pattern may help to identify new cases. In Jiangsu, the mean age of new cases was 60.56 years, and no adolescent cases were identified, which is a great success. Some children presenting with leprosy are considered to be indicative cases (the first case of a family), and the presence of patients <15 years old among new cases represents a deficiency in leprosy control measures (43). In other words, there was no contact transmission within the family to some extent in Jiangsu province. Furthermore, most new cases were detected in skin clinics (68.5%), which reflects hospital practitioners' low sensitivity to the early signs and symptoms of leprosy. The difference in suspicious symptom surveillance reported by dermatology clinics is that it promotes proactive rather than reactive reporting of suspicious cases by dermatologists. Dermatologists play a vital role in the detection of leprosy cases. Efforts were still needed to strengthen the training of dermatologists in healthcare facilities, especially in low-endemic regions.

In this study, we had two main research objectives. The first was to identify areas at high risk of leprosy, improve the efficiency of interventions, and reduce the excessive consumption of health resources; the second was to summarize the results we have achieved in leprosy over the past years and identify gaps in our work. Geographic information systems (GIS) coupled with spatial scan statistics were successfully applied to explore the spatial-temporal aggregation patterns of leprosy. The approach can be used to visualize the three-interval distribution characteristics of leprosy and detect whether the spread of the disease is aggregated, which is impossible to be accomplished by traditional epidemiology. The high-risk areas identified could serve as an essential starting point for future surveillance in resource-limited regions. In addition to providing disease surveillance targets, these high-risk areas could be prioritized in allocating health resources to achieve adequate disease control (G2D or the proportion of MB patients). In contrast to previous studies, we applied new case data over a relatively long period (16 years) to provide more reliable evidence on the regular distribution of the disease (65–68).

Spatial autocorrelation results indicated a significant spatial clustering distribution of leprosy. The high NCDR regions were mainly located in the northwestern areas during 2005–2020. The NCDR varied considerably among cities or counties, primarily representing a lack of effective surveillance in low-endemic and pseudosilent regions. These changes in leprosy prevalence also exacerbate the problem of local underdiagnosis. Local spatial autocorrelation analysis identified four highly correlated clusters. The High-high cluster was mainly concentrated in the northwestern part of Jiangsu Province. The cluster covers four prefecture-level cities and seven counties, with Suqian recording the highest NCDR among the prefecture-level cities and Pizhou among the county-level cities. It is well-known that the economy of the northwestern region of Jiangsu is not as developed as that of the central and southern parts (69). As a result, resource support and health services may not be enough. Therefore, we should increase the investment of medical resources in this high-risk area. Experts indicated that the continued spread of leprosy in these areas was due to the following reasons in addition to poverty: First, the people affected by leprosy prefer to seek initial treatment from the private informal health system rather than the public health system. Second, inadequate contact tracing with family members or the neighborhood in these regions. Finally, lack of awareness of leprosy and insufficient health education. The high-low cluster was concentrated in the southeastern part of the province and primarily consisted of Wuzhong and Xiangcheng. According to a survey conducted in Zhejiang Province (70), two regions (Huzhou and Jiaxing) bordered by Wuzhong have also suffered from leprosy during the study period. Thus, we guess that inter-provincial transmission may occur in these areas. We will conduct further investigations to confirm our views.

With social and economic development, newly discovered cases originating from the mobile population are a new challenge for leprosy control, as they are more difficult to detect than the resident population, which will increase the risk of developing G2D. In Southeast Asia, such as Malaysia and Thailand, more than 39 and 24% of newly detected cases were reported to be foreign-born (71). More than 70% of new cases in Kuwait were from the mobile population (72). As the result indicated, 55 (15.15%) newly detected cases came from other provinces but lived in Jiangsu. Most came from highly endemic areas (Sichuan and Guizhou) (25, 52). These people came to Jiangsu mainly for work, and there was a potential risk of inter-provincial transmission. We can conclude that newly detected leprosy cases originating from floating populations have posed new threats to leprosy control. The lack of awareness of leprosy among these people also contributes to the infection. Therefore, further inter-provincial epidemiological cooperation was needed besides surveillance of high-prevalence areas in Jiangsu Province.

Combined with the results presented, although new cases in Jiangsu have decreased, some regions still suffer from the burden of leprosy. The following points should be given special attention. Firstly, strengthen disease surveillance capacity and provide sustainable financial support to endemic areas (Suqain, Xuzhou, etc.). Secondly, improve the quality of clinical services, including early diagnosis, treatment, and the ability to manage complications. Thirdly, strengthen intervention and rehabilitation services for people with disabilities or impairments to enable them to reintegrate into society. Fourthly, health education is needed to raise awareness of leprosy among high-risk groups (farmers) for early detection and to improve their self-care capabilities. At last, scientific research should be conducted to explore new approaches to control leprosy.

## Conclusion

This study mainly described the epidemiological characteristics of leprosy in Jiangsu Province from 2005 to 2020. As the results indicated, the NCDR of leprosy decreased, but the new cases showed disabilities, a sign of the late diagnosis. Newly detected leprosy cases were spatially concentrated in the northwest province, such as Suqian city. Special attention should be paid to the High-Low cluster gathered in Wuzhong, which was close to the endemic leprosy area in Zhejiang Province and indicated possible inter-provincial transmission. Effective strategies were required to promote leprosy control in these areas. For example, health education for farmers was needed to increase their awareness of leprosy, aiming to achieve early detection and improve their self-care capabilities. There was also an urgent need to strengthen the capacity for early detection and standardized management to achieve the goal of 0 leprosy cases.

## Limitations

The study has some limitations. The classification of MB and PB has been continuously adjusted over the past decades. Different types can affect epidemiological studies. Therefore, comparing work performed 20 years ago with more recent work is difficult. Consequently, we did not combine previous data with the data presented in this paper for comparison. Misclassification is at risk in different countries and even within countries (such as education level). In addition, no valid evidence is available to prove whether cases outside of Jiangsu Province (55 patients) were infected before or after arriving.

## Data availability statement

The data analyzed in this study is subject to the following licenses/restrictions: Data supporting the results of this study are available from the Jiangsu Provincial Center for Disease Control and Prevention; however, the availability of these data is limited, they are used with permission from this study, and they are not publicly available. These data are available to the authors upon reasonable request and with permission from the Jiangsu Provincial Center for Disease Control and Prevention. Requests to access these datasets should be directed to zhanglh@jscdc.cn.

## Ethics statement

Ethical review and approval was not required for the study on human participants in accordance with the local legislation and institutional requirements. Written informed consent for participation was not required for this study in accordance with the national legislation and the institutional requirements.

## Author contributions

XL: methodology, software, formal analysis, and writing. GJ: conceptualization, project administration, and writing. JY: data curation and writing. YL: methodology. LZ and PW: conceptualization, supervision, and writing. All authors contributed to the article and approved the submitted version.
